# A Transient Increase in the Serum ANCAs in Patients with SARS-CoV-2 Infection: A Signal of Subclinical Vasculitis or an Epiphenomenon with No Clinical Manifestations? A Pilot Study

**DOI:** 10.3390/v13091718

**Published:** 2021-08-29

**Authors:** Monica Gelzo, Sara Cacciapuoti, Biagio Pinchera, Annunziata De Rosa, Gustavo Cernera, Filippo Scialò, Marika Comegna, Mauro Mormile, Antonella Gallicchio, Gabriella Fabbrocini, Roberto Parrella, Gaetano Corso, Ivan Gentile, Giuseppe Castaldo

**Affiliations:** 1 CEINGE-Biotecnologie Avanzate, Scarl, 80145 Naples, Italy; gelzo@ceinge.unina.it (M.G.); cernera@ceinge.unina.it (G.C.); filippo.scialo@unicampania.it (F.S.); marika.comegna@unina.it (M.C.); gaetano.corso@unifg.it (G.C.); 2 Dipartimento di Medicina Molecolare e Biotecnologie Mediche, Università di Napoli Federico II, 80131 Naples, Italy; 3 Dipartimento di Medicina Clinica e Chirurgia, Università di Napoli Federico II, 80131 Naples, Italy; sara.cacciapuoti@libero.it (S.C.); biapin89@virgilio.it (B.P.); mormile@unina.it (M.M.); gallicchioantonella9@gmail.com (A.G.); gafabbro@unina.it (G.F.); ivan.gentile@unina.it (I.G.); 4 Dipartimento di Malattie Infettive e Emergenze Infettive, Divisione di Malattie Infettive Respiratorie, Ospedale Cotugno, AORN dei Colli, 80131 Naples, Italy; annunziataderosa@yahoo.it (A.D.R.); rob.parrella@gmail.com (R.P.); 5 Dipartimento di Medicina Traslazionale, Università della Campania L. Vanvitelli, 80131 Naples, Italy; 6 Dipartimento di Medicina Clinica e Sperimentale, Università di Foggia, 71121 Foggia, Italy

**Keywords:** ANCA, MPO, PR3, endothelial damage

## Abstract

A relationship is emerging between SARS-CoV-2 infections and ANCA-associated vasculitis (AAV) because: (i) the pulmonary involvement of COVID-19 may mimic that observed in patients with AAV; (ii) the two diseases may occur together; (iii) COVID-19 may trigger AAV. However, few cases of AAV have been identified so far in COVID-19 patients. To define the frequency of ANCA autoimmunity in patients with SARS-CoV-2 infection, we analyzed the serum ANCAs and the serum PR3 and MPO antigens by immunoassays in 124 adult patients with a diagnosis of SARS-CoV-2 infection (16 were asymptomatic and 108 were hospitalized) and 48 control subjects. The serum ANCAs were significantly higher in the hospitalized patients compared with either the controls or the asymptomatic patients and increased with the progression of the COVID-19 severity. After one week of hospitalization, the values were significantly lower. In contrast, no differences emerged among the controls, asymptomatic and hospitalized patients for the PR3 and MPO serum levels. None of the patients had clinical signs of AAV with the exception of a severe pulmonary involvement. Further studies are necessary to define whether the increase in the serum ANCAs might mask subclinical vasculitis in a percentage of patients with SARS-CoV-2 infection or it is an epiphenomenon of SARS-CoV-2 infection with no clinical manifestations.

## 1. Introduction

Vascular complications have been reported in patients with a SARS-CoV-2 (severe acute respiratory syndrome coronavirus 2) infection. Among these, hypercoagulability [1] with a high occurrence of both arterial and deep vein thromboses with pulmonary embolisms was found in a high percentage of severe cases [1,2]. Necroscopies of COVID-19 patients revealed that endothelial cell inflammation with the accumulation of lymphocytes, plasma cells and monocytes involved several organs [3], particularly the lung [4]. In addition, circulating neutrophils, which are increased in COVID-19 patients [5], contribute to the endothelial damage by releasing tumor necrosis factor-alpha, interleukin (IL)-1 and IL-8.

Acute kidney disease was described in up to one third of severe patients with COVID-19 [6] and may appear within a more complex picture of ANCA (anti-neutrophil cytoplasmic antibody)-associated vasculitis either after or during COVID-19 [7]. ANCA-associated vasculitis (AAV) comprises granulomatosis with polyangiitis (GPA), microscopic polyangiitis and eosinophilic GPA [8]. AAV may be triggered by the release of proteinase 3 (PR3) and myeloperoxidase (MPO) antigens that are stored in the cytoplasmic granules of neutrophils [9]. The degranulation due to neutrophil apoptosis or programmed cell death during inflammation may cause the enhanced exposition of PR3 and MPO, exceeding the ability of specific inhibitors such as alpha-1-antytripsin to degrade these antigens and thus trigger the autoimmune response [9]. Other factors such as genetics, drugs, bacteria and viruses may contribute to the pathogenesis of AAV. Recently, ANCAs have been observed in many other diseases such as autoimmune rheumatic diseases [10], inflammatory bowel diseases [11] and infections [12,13].

In this context, relationships are emerging between COVID-19 and AAV because the pulmonary involvement of COVID-19 may mimic that observed in patients with AAV; furthermore, the two diseases may occur together and, finally, SARS-CoV-2 infection may trigger AAV [7]. However, only a few cases of AAV have been identified so far in patients with a SARS-CoV-2 infection [7]. On the other hand, other autoimmune conditions have also been implicated in the context of COVID-19 such as cold agglutinin syndrome, autoimmune hemolytic anemia and Guillain–Barré syndrome [14].

To define the frequency of ANCA autoimmunity in patients with SARS-CoV-2 infection we studied the serum ANCAs and the serum PR3 and MPO antigens in asymptomatic and in hospitalized COVID-19 patients at hospital admission and one week after the recovery.

## 2. Patients and Methods

### 2.1. Patients

We enrolled 124 adult patients with a diagnosis of SARS-CoV-2 infection. Sixteen patients had an asymptomatic course of the disease up to the virologic recovery. Thirty-five patients were admitted from March to May 2020 (first wave) and 73 patients were admitted from September 2020 to May 2021 (second wave) at either the Department of Clinical Medicine and Surgery, Section of Infectious Diseases, University Hospital Federico II, Naples or the Department of Infectious Disease and Infectious Urgencies, Cotugno Hospital, AORN dei Colli, Naples. The study was approved by the Ethical Committee of the University Hospital Federico II of Naples; the exclusion criteria were either the refusal of or the impossibility to obtain informed consent. The 35 patients of the first wave had a median age of 61 years (interquartile range (IQR): 50–73) and included 8 females (23%). The 73 patients of the second wave had a median age of 35 years (IQR: 30–49) and included 56 females (77%). The diagnosis of a SARS-CoV-2 infection was confirmed by a molecular analysis (RT-PCR) of a nasopharyngeal swab [15]. All the enrolled patients were classified on the basis of the seven ordinal scale created by the World Health Organization (WHO) Research and Development Blueprint expert group used in previous influenza studies. For each patient, we considered the worst WHO stage during the infection [16,17,18]. The frequencies of chronic renal failure in the patients of both waves were less than 5%. All biomarkers were tested at admission for the hospitalized COVID-19 patients and at diagnosis for the asymptomatic ones. Furthermore, we studied 48 control subjects with a median age of 43 years (IQR: 33–61; 21 females, 44%).

### 2.2. Immunoassays

The serum ANCAs were measured by a human ANCA ELISA kit (MyBioSource, Inc., San Diego, CA, USA) without a sample dilution. The serum PR3 was quantified by a human PR3 ELISA kit (Fine test, Wuhan Fine Biotech Co., Ltd., Wuhan, China) by a 1:2 dilution of the samples. The serum MPO and IL-6 were analyzed by a human Magnetic Luminex Assay on a Biorad Bio-Plex 100 system (Labospace s.r.l., Milan, Italy) by 1:50 and 1:2 dilutions of the samples, respectively. The imprecision (CV%) and inaccuracy (%) values of the methods are reported in the Supplementary Materials (Table S1). The analyses were performed at diagnosis in asymptomatic patients and at hospital admission and one week later in hospitalized patients.

### 2.3. Statistical Analysis

Data were reported as a median and an IQR. A Shapiro–Wilk test was used to test the normality of the distributions that were significantly non-normal. The comparisons between two groups were evaluated by a Mann–Whitney U test. The statistical differences between three groups were assessed by a Kruskal–Wallis test and the pairwise comparisons by Mann–Whitney U test. Paired comparisons were performed by a Wilcoxon signed-rank test. The statistical analysis was performed by SPSS (version 26, IBM SPSS Statistics, Chicago, IL, USA)). The graphics were performed by KaleidaGraph software (version 4.5.4, Synergy, Reading, PA, USA). *p*-values < 0.05 were considered to be significant.

## 3. Results

Table 1 shows the levels of the serum ANCAs, MPO and PR3 in the controls and asymptomatic and hospitalized patients with a SARS-CoV-2 infection at admission. The serum levels of ANCAs were significantly higher in hospitalized patients compared with both the controls and asymptomatic patients. The serum levels of MPO were significantly higher in asymptomatic patients compared with the controls. In contrast, we found no difference between the controls and hospitalized patients for MPO or among the controls, asymptomatic and hospitalized COVID-19 patients for PR3. In addition, we tested the serum IL-6, which was significantly (*p* = 0.001) higher in the hospitalized patients (35.9 pg/mL, IQR: 25.7–128 pg/mL) than the controls (26.0 pg/mL, IQR: 19.8–41.9 pg/mL). The IL-6 levels in the asymptomatic patients (26.5 pg/mL, IQR: 19.8–38.4 pg/mL) were not different compared with both the controls and the hospitalized patients.

The conversion factors to SI units (ng/mL × Factor = nmol/m^3^) were 6.67 for ANCAs and MPO and 34.5 for PR3.

**Figure 1:**
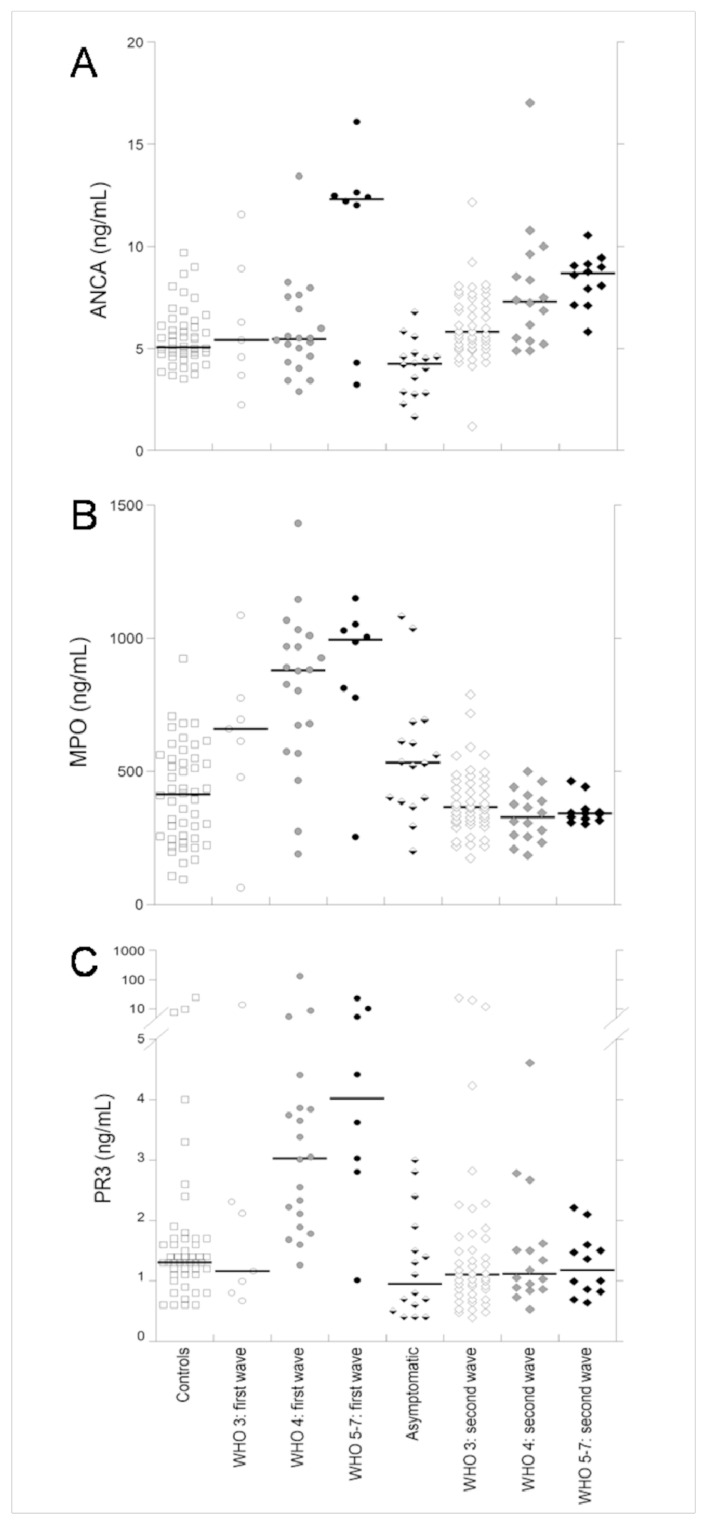
The serum ANCAs (**A**), MPO (**B**) and PR3 (**C**) in the controls, hospitalized COVID-19 patients of the first wave at admission with WHO stages 3, 4 and 5–7, asymptomatic patients with a SARS-CoV-2 infection and hospitalized COVID-19 patients of the second wave at admission with WHO stages 3, 4 and 5–7.

Table 2 shows the comparison of age and the serum ANCAs, MPO and PR3 in hospitalized patients with a SARS-CoV-2 infection of the two waves at admission, classified according to the clinical WHO stage. The age was significantly lower in the patients of the second wave of each stage compared with the patients of the first wave. Furthermore, the age gradually increased (significantly for the patients of the second wave) with the increase in the WHO stage. The ANCA levels (Table 2 and Figure 1A) were not significantly different between the patients with a SARS-CoV-2 infection of the two waves in any of the WHO stages whereas the serum ANCAs gradually increased to a significance with the progression of the stage among the patients of the second wave.

The serum MPO (Table 2 and Figure 1B) was significantly lower in the patients of the second wave of each WHO stage compared with the patients of the first wave. Furthermore, in the patients of the first wave we observed an increasing trend of MPO with the increasing WHO stage (although not significant). In contrast, in the patients of the second wave, the levels of MPO were not different among the three WHO stages.

Finally, the serum levels of PR3 were not different in the patients of the first and second wave of WHO stage 3 whereas they were significantly lower in the patients of the second wave of both WHO stages 4 and 5–7. Furthermore, in the patients of the first wave, the levels of PR3 were significantly increased with the increasing WHO stage. For the patients of the second wave, no differences were observed among the WHO stages.

**Figure 2:**
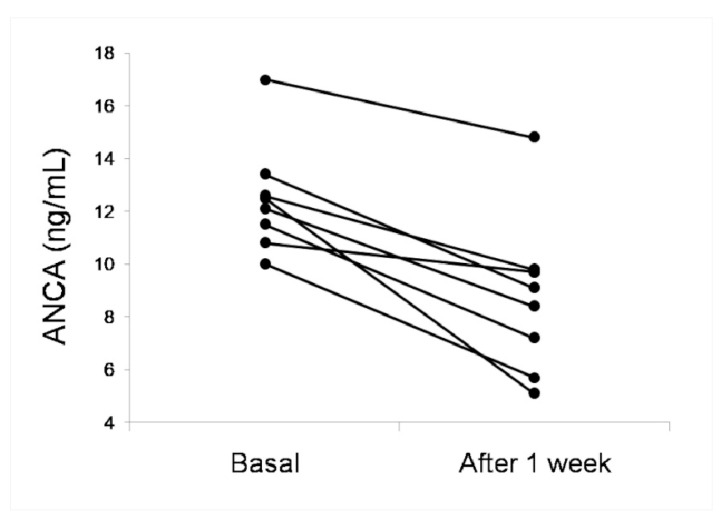
The serum ANCAs in eight hospitalized COVID-19 patients at admission (basal) and after one week of hospitalization.

Table 3 shows the comparison of the serum levels of ANCAs, MPO and PR3 in the hospitalized patients with a SARS-CoV-2 infection at admission (basal) and after one week of hospitalization. The levels of ANCAs decreased in the patients of WHO stage 4 and 5–7 (significantly in the latter). Figure 2 shows the serum levels of ANCAs of 8 hospitalized patients with SARS-CoV-2 infection that had values > 10 ng/mL at admission. In all these patients, the levels decreased after one week of hospitalization. The levels of the serum MPO were not different at admission and after one week of hospitalization in the patients of WHO stages 3 and 4 whereas the values decreased, although not significantly, in the patients of WHO stage 5–7 (Table 3). The PR3 levels significantly increased after one week of hospitalization in the patients of WHO stage 3 whereas they decreased, not significantly, in the patients of stages 4 and 5–7.

## 4. Discussion

COVID-19 patients present with inflammation not only at the respiratory level but also in other districts [3]. Primary systemic vasculitis has varying degrees of inflammation of the blood vessel wall. Severe and rare forms are difficult to diagnose. Furthermore, the recognition of systemic vasculitis associated with autoantibodies against intracellular and tissue antigens has important diagnostic and prognostic implications for the clinical management of patients [8].

Autoantibodies detected in systemic vasculitis are extremely heterogeneous concerning their specificity and clinical significance. Only a few of these autoantibodies, such as ANCAs, are currently used in routine practice in the diagnosis of patients with small-vessel vasculitis [8]. Moreover, in addition to MPO and PR3, other antigens can lead to the production of autoantibodies [19]. Based on this background, we aimed to test the total circulating ANCAs and we observed that patients with a SARS-CoV-2 infection had serum levels of ANCAs that were significantly higher than the control subjects. These levels increased in asymptomatic and hospitalized patients and further raised with the progression of the WHO stage. The production of ANCAs in patients with SARS-CoV-2 infection seems to be triggered by the enhanced release of the PR3 and MPO antigens by neutrophils; in the patients with SARS-CoV-2 infection of the first wave (hospitalized between March and May 2020), the serum levels of PR3 and MPO were parallel to the trend of the serum ANCAs. In the patients of the second wave (hospitalized between September 2020 and May 2021), we observed again an increase in the serum ANCAs but the serum levels of both the MPO and PR3 antigens were normal. Our data agreed with the lower number of circulating neutrophils that we observed in patients with SARS-CoV-2 infection of the second wave compared with those of the first wave and then on the lower release of cytoplasmic antigens during inflammation [5]. Thus, the increase in the ANCAs in the patients of the second wave remains unexplained. This finding could be explained by the release of other antigens that can lead to ANCA production [19]. Furthermore, another explanation might be the formation of neutrophil extracellular traps in which neutrophils kill pathogens. The subsequent macrophage phagocytosis would cause a release and exposition of neutrophil granular proteins (MPO and PR3) with the subsequent production of ANCAs [20]. Lastly, the production of ANCAs may be due to the epitope spreading already described in AAV [21] or it may be due to the mechanisms of autoantigen complementarity or molecular mimicry [22]. It is interesting to note the lack of renal alterations and other symptoms suggestive of AAV in all patients with a SARS-CoV-2 infection that had an increase in the serum ANCAs except for one severe pulmonary involvement requiring oxygen. However, such a pulmonary disease may be also associated with endothelial damage and with vasculitis as it has been previously demonstrated in patients with COVID-19 [4]. Thus, a pathogenic role of ANCAs in patients with SARS-CoV-2 infection is questionable, considering also the rapid decrease in the serum ANCAs observed in our patients after one week of hospitalization. It is possible that the transient increase in the ANCAs observed in our patients with SARS-CoV-2 infection may depend on the reduced affinity maturation of immunoglobulins, which may explain the presence of ANCAs with a lower avidity already described in asymptomatic individuals [23]. A study limitation is the absence of a control group of patients with other infections that could show similar levels of ANCAs and antigens [12,13].

## 5. Conclusions

In most patients with SARS-CoV-2 infection, particularly in the advanced stages, there is an increase in the serum ANCAs. In the patients of the first wave, such an increase seemed to be due to the enhanced release of MPO and PR3 whereas in the patients of the second wave, other mechanisms might be implicated. The increase in the ANCAs was not associated with the typical symptoms of vasculitis except for one case of severe lung disease. Moreover, this increase was transient as the levels of the serum ANCAs, as well as the levels of PR3 and MPO, reduced after one week of hospitalization. Further studies are necessary to define whether the increase in the serum ANCAs might mask a subclinical vasculitis, if it is an epiphenomenon of SARS-CoV-2 infection with no clinical manifestations or if it could be an early autoimmune event with chronic long-term clinical consequences.

## Figures and Tables

**Table 1:** Comparison of age, serum ANCAs, MPO and PR3 in the controls, asymptomatic and hospitalized COVID-19 patients at admission. Median and IQR.

	Controls	Asymptomatic	Hospitalized	Kruskal–Wallis
N	48	16	108	-
Age (years)	43 (33–61)	49 (42–61)	41 (32–61)	n.s.
ANCAs (ng/mL)	5.1 (4.7–6.1)	4.3 (2.8–4.7) ^a^	6.5 (5.0–8.1) ^a,b^	<0.0001
MPO (ng/mL)	413 (249–548)	533 (389–667) ^a^	396 (312–669)	n.s.
PR3 (ng/mL)	1.3 (1.1–1.7)	1.0 (0.5–1.8)	1.4 (0.9–2.6)	n.s.

^a^*p* < 0.01 versus controls; ^b^ *p* < 0.01 versus asymptomatic patients. N.s.: not significant.

**Table 2:** Comparison of age, serum ANCAs, MPO and PR3 in hospitalized COVID-19 patients of the first wave and second wave at admission. Median and IQR.

	Wave	WHO 3	WHO 4	WHO 5–7	Kruskal–Wallis
N	1st	7	20	8	-
	2nd	45	16	12	-
Age	1st	60 (39–62)	64 (51–73)	75 (57–79)	n.s.
(years)	2nd	32 (28–39)	34 (30–49)	51 (44–56) ^a,b^	<0.0001
	1st versus 2nd	0.016	0.001	0.016	
ANCA	1st	5.4 (3.3–9.6)	5.5 (3.9–7.7)	12.5 (3.8–14.4)	n.s.
(ng/mL)	2nd	5.8 (5.0–7.4)	7.3 (5.4–9.3) ^a^	8.7 (7.3–9.1) ^a^	<0.0001
	1st versus 2nd	n.s.	n.s.	n.s.	
MPO	1st	658 (477–775)	878 (598–999)	994 (785–1046)	n.s.
(ng/mL)	2nd	365 (308–459)	327 (255–404)	341 (317–355)	n.s.
	1st versus 2nd	0.008	<0.0001	0.004	
PR3	1st	1.2 (0.8–2.3)	3.0 (1.9–3.9) ^a^	4.0 (2.8–9.1) ^a^	0.041
(ng/mL)	2nd	1.1 (0.8–1.7)	1.1 (0.9–1.6)	1.2 (0.8–1.6)	n.s.
	1st versus 2nd	n.s.	<0.0001	<0.0001	

^a^ *p* < 0.01 versus WHO 3; ^b^ *p* < 0.01 versus WHO 4. N.s.: not significant.

**Table 3:** Comparison of the serum ANCAs, MPO and PR3 in hospitalized COVID-19 patients at admission and after one week. Median and IQR.

		WHO 3 (n = 18)	WHO 4 (n = 24)	WHO 5–7 (n = 10)
ANCA	Basal	6.3 (4.7–7.7)	7.5 (4.9–9.0)	8.1 (7.1–12.6)
(ng/mL)	After 1 week	6.8 (4.8–8.0)	5.7 (4.4–8.9)	6.2 (6.0–6.8)
	*p*-value ^a^	n.s.	n.s.	0.043
MPO	Basal	466 (353–667)	570 (334–898)	794 (323–1034)
(ng/mL)	After 1 week	474 (367–731)	612 (341–958)	551 (362–1161)
	*p*-value ^a^	n.s.	n.s.	n.s.
PR3	Basal	1.3 (0.8–2.3)	2.3 (1.6–3.7)	2.9 (1.4–6.6)
(ng/mL)	After 1 week	1.8 (1.0–2.5)	1.9 (1.2–2.7)	1.7 (1.5–2.2)
	*p*-value ^a^	0.022	n.s.	n.s.

^a^ Wilcoxon signed-rank test. N.s.: not significant.

## Data Availability

The data presented in this study are available on request from the corresponding author.

## Supplementary Materials

The following are available online at https://www.mdpi.com/article/10.3390/v13091718/s1. Table S1: Imprecision (CV%) and inaccuracy (%) values of the analytical methods evaluated on the highest and lower calibration points.
