# Supplementary material for: A Transient Increase in the Serum ANCAs in Patients with SARS-CoV-2 Infection: A Signal of Subclinical Vasculitis or an Epiphenomenon with No Clinical Manifestations? A Pilot Study
Source: Viruses. 2021 Aug 29;13(9):1718. doi: 10.3390/v13091718 (PMC8473224; doi:10.3390/v13091718)
Supplement: Supplementary file 1 [file viruses-13-01718-s001.zip › viruses-1321980-supplementary.pdf]

Table S1. Imprecision (CV%) and inaccuracy (%) values of analytical methods evaluated on the highest and lower calibration points.

	Imprecision (CV%)	Inaccuracy (%)
ANCA (ng/mL)		
20	0.07	−0.10
0.62	0.29	14.5
MPO (ng/mL)		
29.7	3.74	0.28
0.04	11.0	−10.7
PR3 (ng/mL)		
10	3.6	0.05
0.16	1.1	−0.11
